# Single-Cell and Bulk Transcriptomics Reveal an Epithelial *LTF*-*LRP11* Signaling Axis Associated with Severe COVID-19 Susceptibility

**DOI:** 10.3390/genes17080982

**Published:** 2026-08-21

**Authors:** Ana Luiza Labbate Bonaldo, Jeferson dos Santos Souza, Jakeline Santos Oliveira, Amanda Piveta Schnepper, Caio Fernando Ferreira Mussatto, Victória Larissa Schimidt Camargo, Paula Paccielli Freire, Otavio Cabral-Marques, Sarah Santiloni Cury, Robson Francisco Carvalho

**Affiliations:** 1 Department of Cellular and Molecular Biology, Institute of Biosciences, São Paulo State University (UNESP), Botucatu 18618-689, SP, Braziljeferson.s.souza@unesp.br (J.d.S.S.);; 2 Department of Immunology, Institute of Biomedical Sciences, University of São Paulo, São Paulo 05508-000, SP, Brazil; 3 Department of Clinical and Toxicological Analyses, School of Pharmaceutical Sciences, University of São Paulo, São Paulo 05508-000, SP, Brazil; 4 Network of Immunity in Infection, Malignancy, Autoimmunity (NIIMA), Universal Scientific Education and Research Network (USERN), São Paulo 05508-000, SP, Brazil; 5 Department of Medicine, Division of Molecular Medicine, University of São Paulo School of Medicine, São Paulo 01246-903, SP, Brazil; 6 Laboratory of Medical Investigation 29, University of São Paulo School of Medicine; São Paulo 01246-903, SP, Brazil

**Keywords:** COVID-19, ligand and receptor, *LTF*, *LRP11*, scRNA-Seq, secretome

## Abstract

Background/Objectives: The molecular mechanisms underlying susceptibility to severe COVID-19 remain incompletely understood. We aimed to identify the signaling pathways associated with disease severity by integrating transcriptomic data and characterizing ligand–receptor interactions involved in the host response to SARS-CoV-2 infection. Methods: We integrated publicly available bulk RNA-sequencing data from nasopharyngeal (NP) swabs (GSE152075) and single-cell RNA-sequencing data from bronchoalveolar lavage fluid samples (GSE145926). Analyses focused on secreted ligands and their cognate receptors and were performed in relation to demographic and clinical characteristics associated with susceptibility to severe COVID-19, including sex, age, and viral load. Results: Patients with characteristics associated with increased susceptibility to severe disease, including male sex, advanced age, and high viral load, exhibited transcriptional programs enriched for inflammatory and immune-response pathways. In contrast, individuals with lower susceptibility displayed reduced expression of 43 ligand genes compared with matched negative controls, suggesting distinct secretory programs associated with the host response to infection. We identified an association between the expression of lactoferrin (*LTF*) and its receptor, LDL receptor-related protein 11 (*LRP11*), and susceptibility to severe COVID-19. *LRP11* was predominantly expressed in human pulmonary epithelial cells, and its expression increased during SARS-CoV-2 infection in monkeys. Conclusions: Our findings provide insight into the molecular mechanisms associated with susceptibility to severe COVID-19 through the analysis of ligand and receptor expression in nasopharyngeal swabs and bronchoalveolar lavage fluid samples. The association between *LTF* and *LRP11* highlights a potentially relevant signaling axis in disease pathogenesis and provides a rationale for future functional studies aimed at clarifying its role in COVID-19 severity.

## 1. Introduction

COVID-19, first confirmed in Wuhan, China [1], has quickly spread across the globe, causing more than six million deaths (“COVID-19 Map—Johns Hopkins Coronavirus Resource Center”, s.d.). SARS-CoV-2 is the etiological agent of COVID-19 and a member of the *Coronaviridae* family of the Betacoronavirus genus, along with SARS-CoV and MERS-CoV [1]. Severe COVID-19 has been associated with certain groups, such as males, those of advanced age, and individuals with high viral loads [2,3,4,5]. Advanced age has also emerged as a significant risk factor for COVID-19 severity, likely due to age-related declines in immune function and increased comorbidities, which are recognized risk factors [2,5,6,7]. Likewise, patients with high viral loads have an elevated risk of developing severe disease, potentially reflecting increased viral replication and associated dysregulation of the host immune response [4,8,9].

Some patients respond to this infection with excessive production of pro-inflammatory cytokines, an event known as a cytokine storm, leading to generalized lung inflammation [10]. This uncontrolled inflammatory cascade can damage host tissues and contribute to disease progression [11,12,13]. The inflammatory milieu can influence the secretion of various proteins and factors, collectively known as the secretome [14]. These secreted molecules, when coding for ligand proteins, can interact with cell surface receptors that play essential roles in intercellular communication, modulating the host immune response, and influencing COVID-19 outcomes and severity. Thus, investigating the secretome in nasopharyngeal (NP) swabs and in bronchoalveolar lavage fluid (BALF) holds promise for identifying key players involved in COVID-19 pathogenesis and severity.

One approach to comprehensively analyze the secretome is RNA sequencing (RNA-Seq). This robust and high-throughput method enables the assessment of the transcriptional profile of an organism or even individual cells [15]. The availability of vast amounts of publicly accessible RNA-Seq data [16,17] facilitates exploration of the secretome and its potential implications across various biological processes and diseases, including COVID-19.

We aimed to identify differentially expressed secretome genes associated with an increased risk of severe COVID-19. Our study revealed 43 secretome genes with decreased expression in patient groups less vulnerable to severe COVID-19 (younger age, female sex, and lower viral load). Among these genes, we focused our subsequent analyses on the lactoferrin (*LTF*) gene because its receptor, *LRP11* (LDL receptor-related protein 11), also showed reduced expression in NP swab samples from patients with low viral load. Using single-cell RNA-seq analysis, we identified the expression of the *LRP11* receptor gene in human lung epithelial cells, which are the primary target cells for SARS-CoV-2 [18,19]. These findings contribute to identifying mediators of COVID-19 development and severity.

## 2. Methods

### 2.1. Data Selection and Differential Expression Analysis of RNA-Seq Data

We selected an RNA-Seq study of nasopharyngeal (NP) swabs from 430 COVID-19-positive and 54 negative patients (GSE152075) (Supplementary Table S1) [4]. The count data were obtained from the Gene Expression Omnibus (GEO) database (https://www.ncbi.nlm.nih.gov/geo/, accessed on 1 March 2021). The Network Analyst 3.0 platform was used to identify differentially expressed genes (DEGs) using the following parameters: log2 fold change ≥ |1.5| and *p*-value ≤ 0.05. We used log2 counts per million (CPM) to normalize the data and LIMMA for statistical analysis. The individuals were separated according to age, following the World Health Organization classification (young, <60 years; elderly, >60 years), sex (women and men), and viral load (high, N1 Ct < 24; low, N1 Ct > 24), according to the cycle threshold (Ct) values of RT-qPCR analyses [3]. Each group of patients was analyzed alongside the respective negative healthy samples, and comparisons in each analysis followed the nomenclature in Table 1. According to the literature [2,5,7,8,9,20,21], SARS-CoV-2-positive patients in the male, older, and high-viral-load groups were considered to have increased susceptibility to severe COVID-19 compared with their respective healthy control individuals. In contrast, SARS-CoV-2-positive patients in the female, young, and low-viral-load groups were considered to have low susceptibility to severe COVID-19.

### 2.2. Transcriptome-Based Secretome Analysis

Human secretome analysis was performed by filtering the DEGs obtained for each comparison (female, male, young, old, low viral load, and high viral load) using the secretome gene list from The Human Protein Atlas [22]. The 2641 human secretome proteins were predicted by a proteome scan using at least two of the following three signal peptide prediction methods: SignalP4.0, Phobius, and SPOCTOPUS.

### 2.3. Analysis of Functional Enrichment

The online tool EnrichR [23,24,25] was used for functional enrichment analysis. The WikiPathways and GO Biological Process databases were used to determine the enriched pathways and ontologies. Values of p-adj < 0.05 were ranked from highest to lowest, and the TOP 5 terms were considered for the list of DEGs filtered by the secretome in each condition.

### 2.4. Interactome Analysis Using RNA-Seq Data

We obtained the Benjamini–Hochberg False-Discovery Rate [FDR] < 0.33 by evaluating the statistical significance of each interaction score to reduce potential false negatives or false positives in our analyses (calculated by multiplying ligand × 0.33 and receptor × 0.33 by the Log10Padj value < 0.05, where a score of 1 for communication would be considered positive). We also employed analysis strategies provided by Armingol et al. [26], calculating the product of the ligand–receptor expression threshold and the receptor–ligand expression in the respective protocols analyzed, with the communication score defined as the product of the fold-change values of genes predicted as ligands and receptors (LXR), respectively.

### 2.5. Protein–Protein Interaction Network (PPI)

The proteins identified in the interactome were submitted to the Search Tool for Retrieval of Interacting Genes (STRING, version 11.5) [27] to construct a PPI network of genes potentially associated with the illness prognosis. To build the network, we considered experiments, databases, co-expression, neighborhood, and co-occurrence as sources of active interactions. The minimum required interaction score was 0.700 (high confidence), and nodes disconnected from the network were hidden to simplify PPI network visualization. To visualize and annotate the PPI, we used Cytoscape v3.8.2 software (Cytoscape: A Software Environment for Integrated Models of Biomolecular Interaction Networks).

### 2.6. Obtaining scRNA-Seq Data from Patients with COVID-19

The selected single-cell sequencing dataset (GSE145926) [28] contained 12 BALF samples from six severe- and three moderate-COVID-19 patients and three healthy controls. The severity of illness was defined as moderate or severe, according to the “COVID-19 Diagnosis and Treatment Protocol” by the China National Health Commission issued on 3 March 2020, described by Liao et al. [28]. A healthy BALF sample (GSM3660650) from the GSE128033 dataset was also added, totaling 13 samples with single-cell RNA-sequencing data.

### 2.7. scRNA-Seq Data Analysis

The demultiplexing of cluster A genes (*LTF*, *MST1*, *SLIT1*, *GPR162*, *LRP1*, *LRP11*, *MST1R*, *GPC1*, and *SDC1*), barcode processing, and single-cell 5′ unique molecular identifier (UMI) were performed using the Cell Ranger Software Suite (v.3.1.0) [29]. Alignment of SARS-CoV-2 and human transcriptome reads in individual cells was performed using the STAR tool [30], which integrates the customized genome comprising the human (GRCh38) and total virus (GenBank: MN908947.3) genomes. Single-cell analysis was performed using R with the Seurat single-cell analysis development package (v.3) [31]. We performed uniform manifold approximation and projection (UMAP) to identify cell clusters. To evaluate the gene expression profile, dot and violin plots were generated from the Seurat package (v. 3) using the dot plot and VlnPlot functions, respectively.

### 2.8. Validation of Selected Genes in COVID-19 Infection Time Course

For the temporal analysis of the gene expression profile of the targets chosen after SARS-CoV-2 infection, we used data from the study by Aid et al. [32] (GSE156701, available from GEO). This study collected BALF transcriptome data on days 1, 2, 4, 7, 10, and 14 in nine adult rhesus macaques infected with SARS-CoV-2. RNA-Seq data were generated by the Illumina NextSeq 6000 platform, as described in the original work [32]. The same methodology used to analyze the GSE152075 dataset was used to identify the DEGs.

### 2.9. Data Analysis and Representation

Bar graphs were constructed using GRAPHPAD PRISM (GRAPHPAD Software v8). The principal component analysis (PCA) was created using the ClustVis online tool (https://biit.cs.ut.ee/clustvis/, accessed on 1 March 2021) [33]. Genes with increased and decreased expression were visualized using Volcano plots generated with the VolcaNoseR tool (http://huygens.science.uva.nl/VolcaNoseR, accessed on 1 March 2021) [34]. The UpSet graph, which visualizes gene intersections with expression changes across the comparisons, was generated using the Intervene tool (http://intervene.shinyapps.io/intervene, accessed on 1 March 2021). Heatmaps were generated using Morpheus software (https://software.broadinstitute.org/morpheus, accessed on 1 March 2021) [35]. A heatmap with k-means clustering was used for clustering.

## 3. Results

### 3.1. Secretome Genes Revealed Distinct Expression Profiles in COVID-19 Patients with Different Susceptibilities to Disease Severity

COVID-19 patients were categorized by sex (female, male), age (young, <60 years; old, >60 years), viral load (high, N1 Ct < 24; low, N1 Ct > 24), and positive COVID-19 diagnosis (Supplementary Figure S1A). Our list of differentially expressed genes (DEGs) from each comparison was filtered using the list of 2641 secreted proteins available from The Human Protein Atlas (Supplementary Table S2). We identified 927 secretome genes that were differentially expressed in at least one comparison (log2 fold change |≥1.5| and p-adj ≤ 0.05) (Supplementary Tables S3–S9). Heatmap and cluster (k-means) analysis revealed distinct gene expression profiles for each group compared with controls (Supplementary Figure S1B).

**Figure 1:**
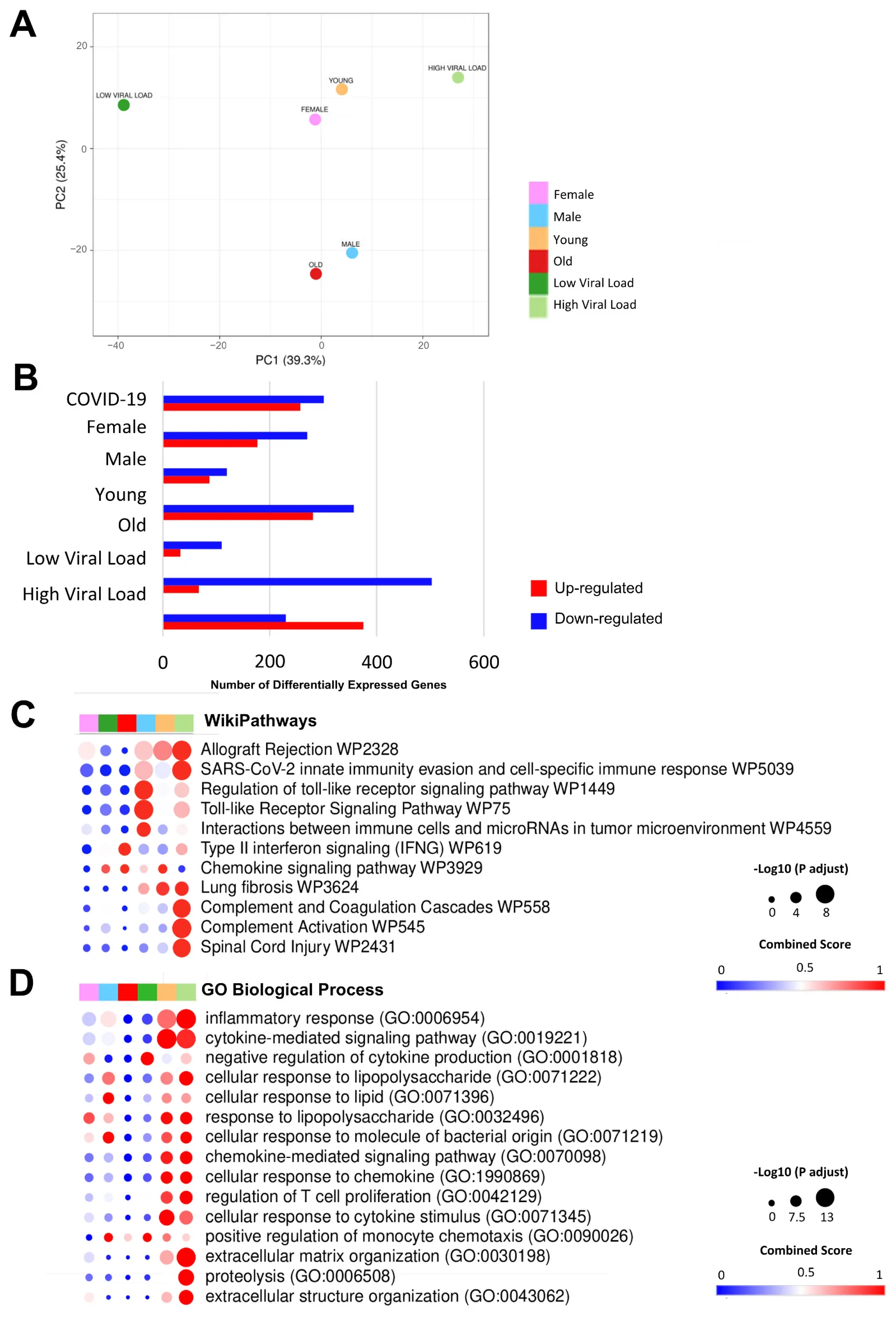
Analysis of secretome gene expression profiles in NP swab samples from COVID-19 patients compared with control individuals by age, sex, and viral load. (**A**) Principal component analysis of the secretome genes. The dark purple dot represents all COVID-19 samples combined. (**B**) Number of DEGs with increased and decreased expression in each comparison. (**C**) Bubble heatmap of the top 5 pathways using the combined score from the WikiPathways and (**D**) GO Biological Process libraries. Bubble size and color intensity represent the adjusted *p*-value, transformed to log10, and the combined score generated by the EnrichR tool, respectively. PC1: First principal component; PC2: Second principal component. WP: WikiPathways identifier; GO: Gene Ontology identifier.

Principal component analysis (PCA) revealed that males, old-age patients, low-viral-load patients, high-viral-load patients, and all COVID-19 patients were distinguishable from females and young patients (Figure 1A). The high-viral-load comparison showed a greater amplitude of fold change (FC|> 1.5|, corrected by Z-Score) of the secretome profile (Figure 1A). Regarding the number of genes with altered expression in the secretome, we observed that men and older adults had fewer DEGs (Figure 1B). We also selected the top 10 DEGs with the most significant variation in expression for each comparison (Supplementary Figure S2A–G). *CXCL9* and *CXCL10* showed increased expression in all groups except the low-viral-load group.

### 3.2. Secretome Genes Are Associated with Inflammatory Pathways and Immune Response to SARS-CoV-2 Infection in Patients with a Higher Risk of Developing Severe COVID-19

Differentially expressed secretome genes for comparisons between COVID-19 patients and control individuals, categorized by age, gender, and viral load, were selected for functional enrichment analysis using the EnrichR [23,24,25] platform with WikiPathways (Figure 1C) and GO Biological Process (Figure 1D) libraries. We found an enrichment of pathways and ontological terms related to immune response and inflammation (Figure 1C,D). Females and patients with a low viral load exhibited lower enrichment of immune responses and inflammation (Figure 1C,D). Patients with a high viral load had enriched pathways and ontologies related to cytokine response, such as the cytokine-mediated signaling pathway, chemokine signaling pathway, and cellular response to cytokine stimulus (Figure 1C,D). We also identified an enrichment of immune and inflammatory response pathways in these patients, including SARS-CoV-2 innate immunity evasion, cell-specific immune responses, inflammatory responses, complement activation, and regulation of T-cell proliferation (Figure 1C,D). Excessive production of pro-inflammatory cytokines, known as a cytokine storm, is commonly associated with an unfavorable prognosis in COVID-19 [13]. We observed that patients with high viral load showed increased expression of pathways related to inflammation, cytokine- and chemokine-mediated signaling, and SARS-CoV-2-specific cellular immune responses. The pro-inflammatory interleukins IL-1, IL-6, IL-17, and tumor necrosis factor-alpha (TNF-α) play an essential role in lung damage in COVID-19 by compromising the respiratory epithelium [12]. In addition, we observed that the same pathways were associated with reduced expression in women. Freire et al. [3] found more robust immune modulation in female patients under 60 years of age, which may explain their favorable prognosis, as well as decreased transcriptional levels of neutrophil-related pro-inflammatory genes, such as CXCL8 and IL1B, compared with male patients [3]. These findings align with other studies and help explain why patients with high viral loads and male individuals are more susceptible to developing severe COVID-19 [8,9,20,36].

### 3.3. Forty-Three Secretome Genes Show Common Differential Expression in Patients with Lower Vulnerability to Develop Severe COVID-19

**Figure 2:**
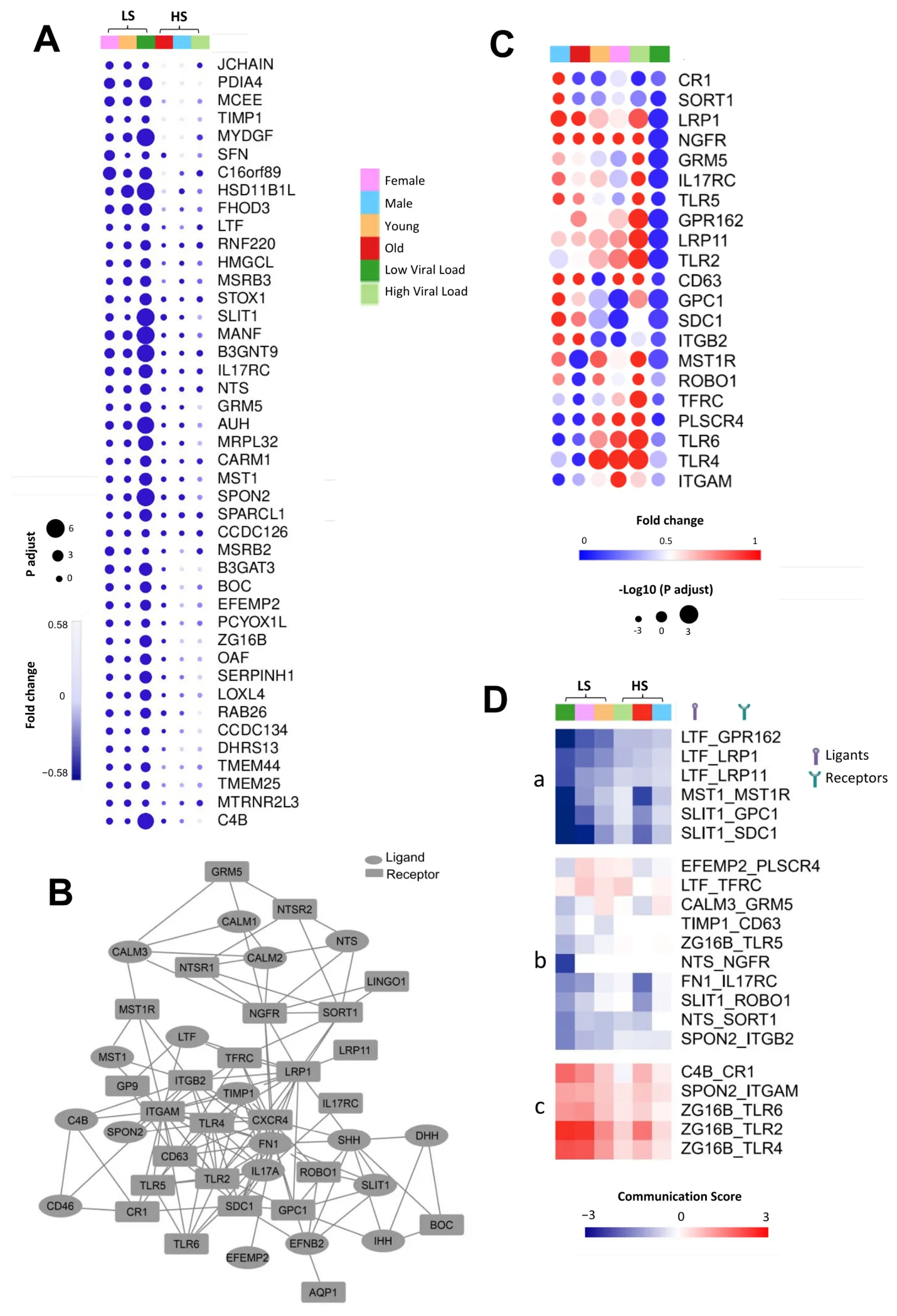
Characterization of 43 secretome genes showing common differential expression in COVID-19 patients with low vulnerability to develop severe disease (female, young, and low viral load). (**A**) Bubble heatmap of 43 genes unique to patients with low susceptibility to develop severe COVID-19. Bubble color intensity represents the adjusted *p*-value (transformed to −log10(P-adjust)), whereas bubble size represents the fold change. Clustering was performed using Euclidean distance. (**B**) Interactome of 18 ligands out of 43 secretome genes and their respective 21 receptors. (**C**) Bubble heatmap showing the expression of the 21 receptors in each group. Bubble color intensity represents fold change, while bubble size represents the adjusted *p*-value, transformed to −log10(P-adjust). (**D**) Heatmap of the interactome based on ligand genes and their respective receptor genes (communication score > 3). Blue represents genes with decreased expression, whereas red represents genes with increased expression. The darker the color, the higher the communication score value of the ligand–receptor pair. Clustering was performed using Euclidean distance. LS: lower susceptibility to severe COVID-19; HS: higher susceptibility to severe COVID-19.

We calculated the number of differentially expressed secretome genes that overlapped among the analyzed comparisons using the UpSet Plot tool (Supplementary Figure S1A). Individuals with the highest susceptibility to severe COVID-19 (older adults, men, and those with a high viral load) did not exhibit overlapping genes (Supplementary Figure S1C). In contrast, the groups with lower susceptibility to severe COVID-19 (women, young people, and those with low viral load) shared 43 genes (Supplementary Figure S1C; Supplementary Table S10). Interestingly, all 43 genes showed greater reductions in patients less vulnerable to severe disease than in those more predisposed to severe disease (Figure 2A). Moreover, many genes showed the most significant statistical significance in patients with low viral loads (Figure 2A).

Investigating ligand and receptor genes holds significant promise for elucidating the molecular mechanisms underlying susceptibility to severe disease. Thus, we constructed a ligand–receptor interaction network using the 43 differentially expressed secretome genes to identify potential signaling interactions (Figure 2B). Of these, 18 genes were identified as ligands interacting with 21 cognate receptors. The network revealed that ligands including *LTF*, *SLIT1*, *ZG16B*, *SPON2*, and *NTS* interacted with multiple receptors, suggesting their potential involvement in diverse signaling pathways (Figure 2B). We next analyzed the expression of the corresponding receptor genes, which were consistently downregulated in patients with low viral loads (Figure 2C). Notably, several of these receptors have been implicated in immune regulation and coagulation-related processes, in agreement with the well-established hypercoagulable state associated with severe COVID-19 [37,38]. Integration of ligand and receptor expression profiles identified three distinct clusters of ligand–receptor pairs (Figure 2D). Candidate ligand–receptor pairs were not prioritized by the number of predicted interactions but rather by their coordinated communication patterns and biological relevance. Among these, cluster A (*LTF-GPR162*, *LTF-LRP1*, *LTF-LRP11*, *MST1-MST1R*, *SLIT1-GPC1*, and *SLIT1-SDC1*) consistently exhibited lower communication scores in patients with lower susceptibility to severe COVID-19, suggesting the coordinated regulation of a signaling module associated with disease severity. Furthermore, lactoferrin (LTF) has been extensively reported to exert antiviral and immunomodulatory activities and has previously been associated with COVID-19 severity [39,40,41,42]. Therefore, based on the coordinated communication pattern observed in cluster A together with the established biological relevance of LTF, we selected this cluster for subsequent analyses.

### 3.4. LRP11 Expression Increases with SARS-CoV-2 Viral Load and COVID-19 Progression

**Figure 3:**
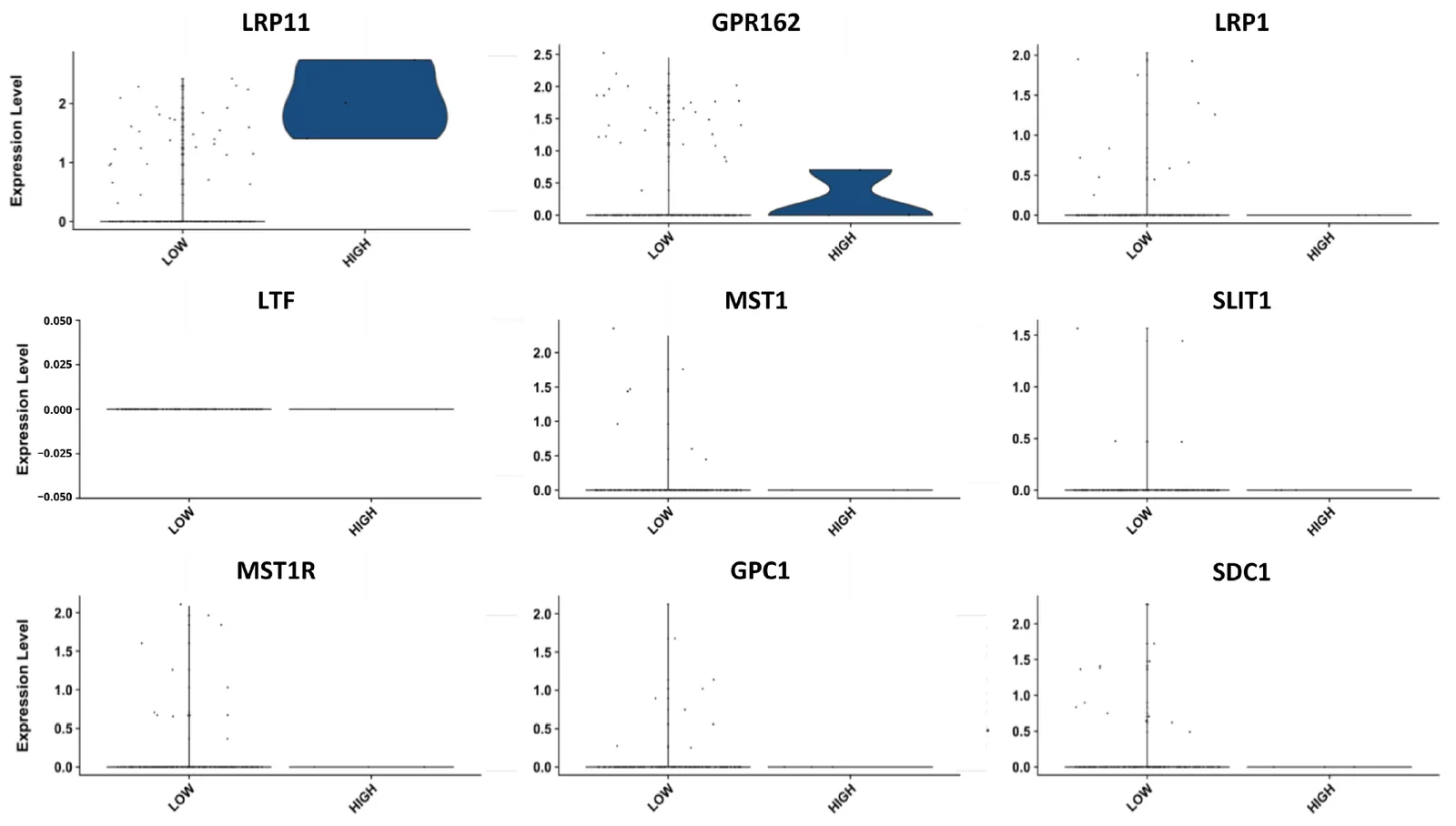
The violin plot illustrates the expression levels of ligands (*LTF*, *MST1*, and *SLIT1*) and their corresponding receptor (*GPR162*, *LRP1*, *LRP11*, *MST1R*, *GPC1*, and *SDC1*) genes from cluster A obtained through single-cell RNA-sequencing analysis of the GSE145926 dataset with an additional healthy BALF sample GSM3660650 (GSE128033 dataset). The *LTF* and *SLIT1* genes were present in more than one ligand–receptor pair but were represented once. High: Patients with high viral load in BALF samples. Low: Patients with low viral load in BALF samples. Among the nine genes shown, only *LRP11* displayed a statistically significant difference in expression between the high- and low-viral-load groups, supporting the specificity of this association.

To further assess the expression levels of the ligand genes (*LTF*, *MST1*, and *SLIT1*) and their corresponding receptor genes (*GPR162*, *LRP1*, *LRP11*, *MST1R*, *GPC1*, and *SDC1*) from cluster A, we conducted scRNA-Seq re-analysis using dataset GSE145926, which includes a healthy sample (GSM36600650) for reference, where the authors characterized the immune cells present in the BALF of patients with varying severity for COVID-19 [28] (Figure 3). Remarkably, consistent with our previous findings, we observed that the LRP11 gene exhibited increased expression in lung epithelial cells from patients with high viral loads, whereas expression was notably decreased in those with low viral loads (Figure 3). The expression levels of the other genes did not differ significantly between patients with high and low viral loads (Figure 3).

**Figure 4:**
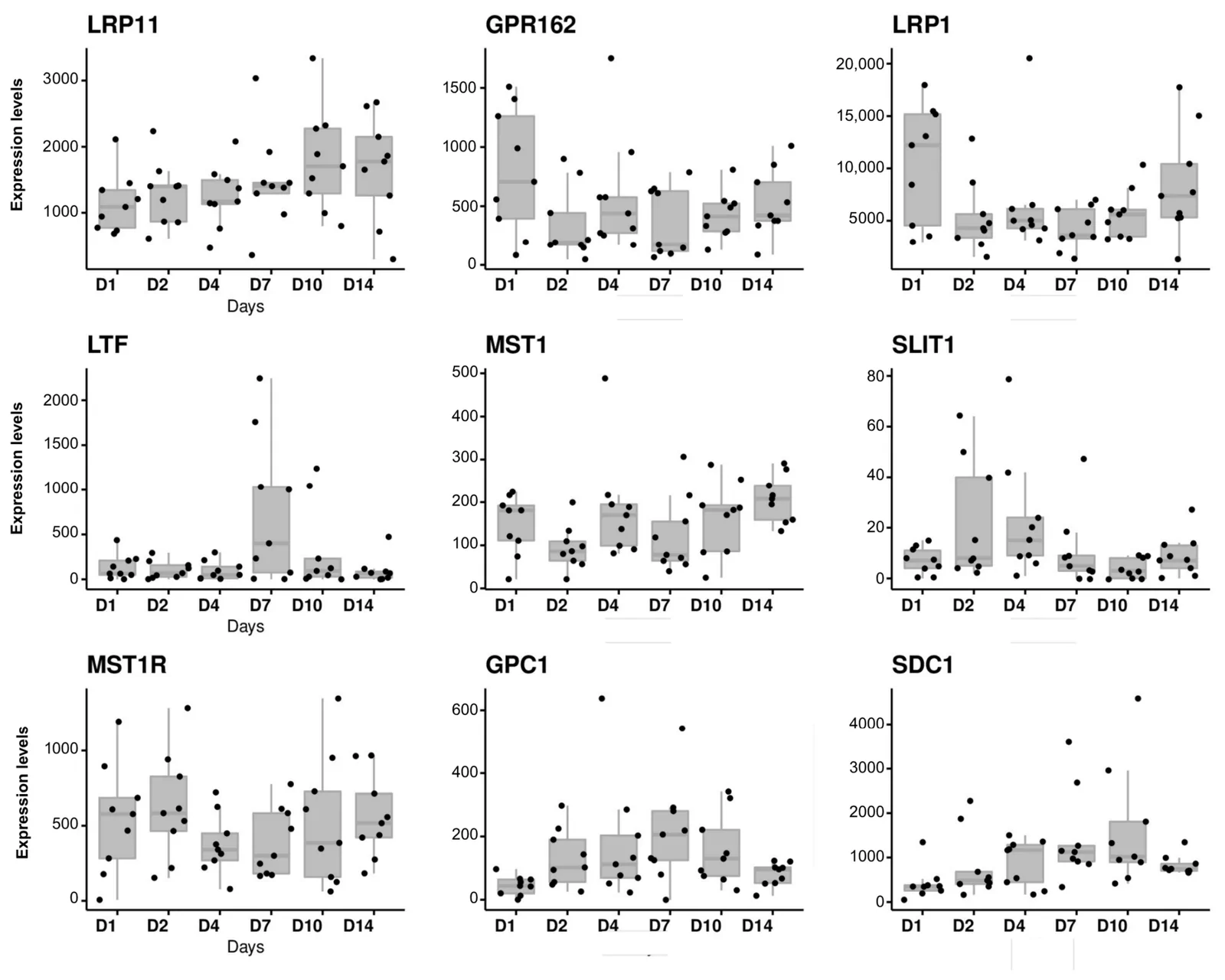
The box plot shows the expression levels of ligand and receptor genes in cluster A of BALF samples from nine adult rhesus macaques during SARS-CoV-2 infection (GSE156701). BALF samples from each infected monkey were collected on days 1, 2, 4, 7, 10, and 14 post-infection. The *LTF* and *SLIT1* genes were present in more than one ligand–receptor pair but were represented once. D1: Day 1; D2: day 2; D4: day 4; D7: day 7; D10: day 10; and D14: day 14.

To confirm our findings, we correlated the gene expression levels of the ligands (*LTF*, *MST1*, and *SLIT1*) and their corresponding receptor genes (*GPR162*, *LRP1*, *LRP11*, *MST1R*, *GPC1*, and *SDC1*) with COVID-19 progression, based on a reanalysis of data from Aid et al. (GSE156701) [32]. These authors evaluated endothelial disruption and vascular thrombosis in rhesus monkeys infected with SARS-CoV-2 [32]. We observed that *LRP11* gene expression in BALF and viral load increased over the post-infection days (days 1, 2, 4, 7, 10, and 14) (Figure 4, Supplementary Table S11). Among the nine genes encoding ligands or receptors, only *LRP11* showed a progressive increase in expression associated with infection progression (Figure 4).

### 3.5. LRP11 Exhibits Epithelial-Enriched Expression and Emerges as a Candidate Receptor Within the Predicted LTF Signaling Axis

**Figure 5:**
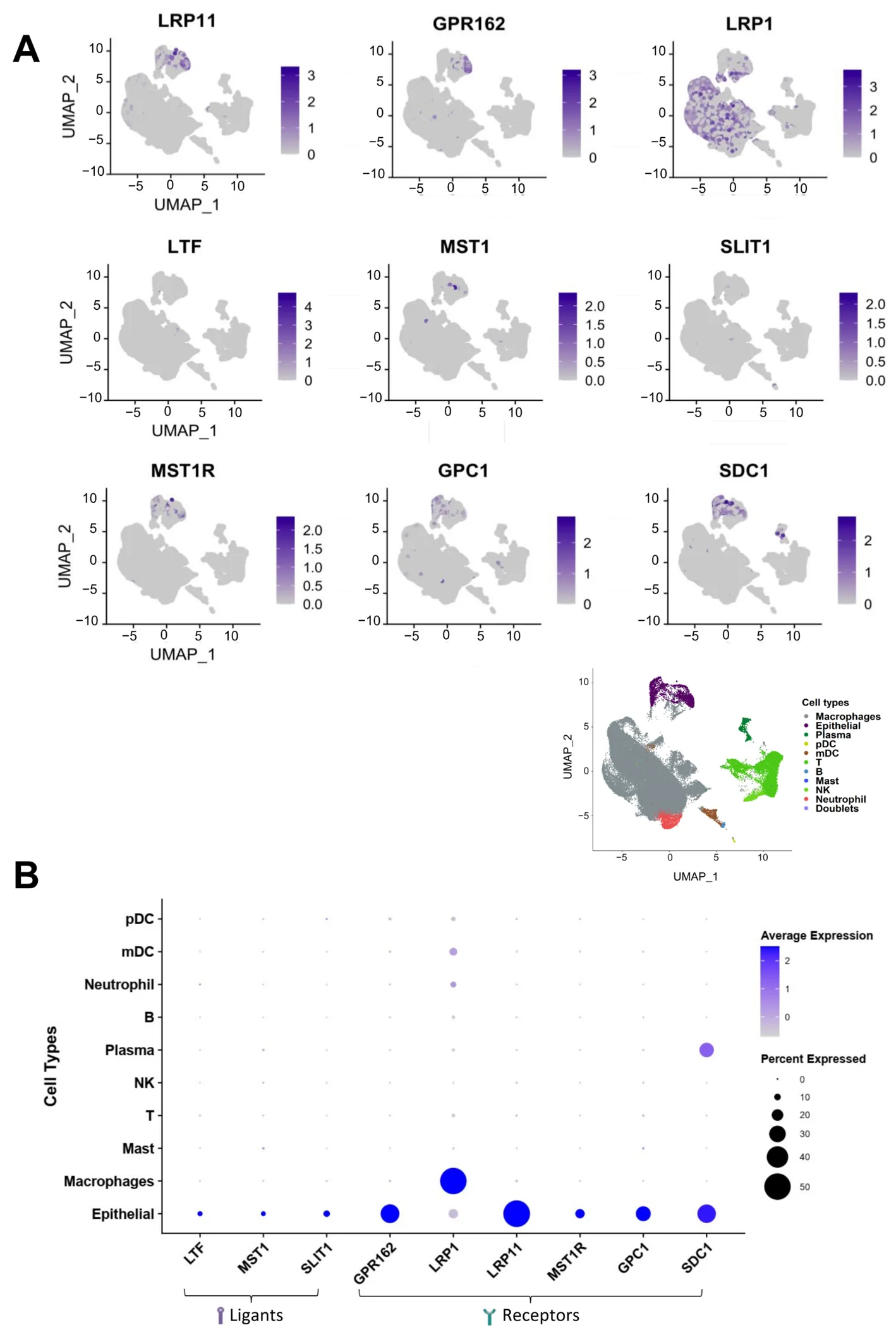
Single-cell expression analysis of ligand genes (*LTF*, *MST1*, and *SLIT1*) and their corresponding receptor genes (*GPR162*, *LRP1*, *LRP11*, *MST1R*, *GPC1*, and *SDC1*) in BALF cell types (GSE145926). (**A**) UMAP plot depicting the distribution of cluster A ligand and receptor expression across cell types in BALF. The sidebars indicate gene expression levels, with darker purple representing greater expression in the cell type corresponding to that zone. The respective cell types were categorized into 11 clusters, each represented by a distinct color. (**B**) The dot plot shows ligand and receptor expression in cluster A for each BALF cell type. The color intensity and dot size represent the average expression of each gene and the percentage expressed in each cell type, respectively.

To characterize the cellular distribution of the ligands (*LTF*, *MST1*, and *SLIT1*) and their corresponding predicted receptors (*GPR162*, *LRP1*, *LRP11*, *MST1R*, *GPC1*, and *SDC1*), we reanalyzed a publicly available single-cell RNA-sequencing (scRNA-seq) dataset comprising bronchoalveolar lavage fluid (BALF) samples from 13 individuals, including six patients with severe COVID-19, three with moderate COVID-19, three healthy controls, and one additional healthy BALF sample. *GPR162* expression was predominantly detected in epithelial cells, whereas *LRP1* showed broad expression across macrophages (Figure 5A), consistent with its well-established role as a multifunctional member of the low-density lipoprotein receptor (LDLR) family [43]. In contrast, LRP11 exhibited a more restricted expression pattern, being predominantly detected in airway epithelial cells—the primary targets of SARS-CoV-2 infection—with lower expression observed in macrophages (Figure 5B).

To determine whether this expression pattern was unique within the LDLR family, we compared the expression profiles of all 13 LDLR family members. Among these receptors, *LRP11* stood out in airway epithelial cells in terms of both average expression and the proportion of *LRP11*-expressing cells (Supplementary Figure S3). We next examined the expression patterns of the ligand genes (*LTF*, *MST1*, and *SLIT1*) and their corresponding receptor genes (*GPR162*, *LRP1*, *LRP11*, *MST1R*, *GPC1*, and *SDC1*). Overall, these genes were predominantly expressed in epithelial cells and macrophages (Supplementary Figure S4). Notably, *LRP11* was expressed in 70% of epithelial cells, compared with 22% of macrophages, 6% of T cells, and only 1% of natural killer (NK) cells and myeloid dendritic cells (mDCs) (Supplementary Figure S4). Based on its marked epithelial enrichment, its distinct expression pattern relative to the broader macrophage distribution of *LRP1*, and its position within the predicted *LTF* signaling axis, *LRP11* was selected for subsequent analyses.

## 4. Discussion

Despite substantial advances in characterizing host transcriptional responses to SARS-CoV-2 infection, the molecular mechanisms associated with susceptibility to severe COVID-19 remain incompletely understood. Severe COVID-19 has been consistently associated with a higher mortality rate in men [20,36], older adults (≥60 years) [2,6,7], and patients with a high viral load [9]. Integrating secretome profiling with predicted ligand–receptor relationships may reveal coordinated intercellular communication programs associated with factors linked to severe COVID-19 susceptibility [14,26]. In this study, we integrated bulk RNA-sequencing data from NP swabs with single-cell RNA-sequencing data from bronchoalveolar lavage fluid and temporal transcriptomic data from SARS-CoV-2-infected rhesus macaques to investigate secretome genes and their predicted receptor interactions in relation to these susceptibility-associated characteristics. This integrative analysis identified distinct secretory and ligand–receptor expression patterns across patient groups and highlighted an epithelial *LRP11*-associated signaling program consistently linked to viral load and disease progression across independent datasets. These findings provide a transcriptomic framework for prioritizing candidate intercellular communication mechanisms for subsequent functional investigation.

Our principal finding was the identification of a convergent secretome signature associated with lower susceptibility to severe COVID-19. Whereas patients with higher susceptibility (males, older adults, and those with high viral load) exhibited heterogeneous transcriptional responses without a common secretome signature, individuals with lower susceptibility (females, younger adults, and those with low viral load) consistently shared a set of 43 secretome genes showing reduced expression relative to their respective comparison groups. The convergence of this transcriptional signature across three independent susceptibility-associated factors suggests that these genes constitute a coordinated molecular program rather than isolated transcriptional changes, providing a robust foundation for subsequent ligand–receptor interaction analyses. Collectively, these findings indicate that common intercellular communication pathways may underlie the differential host responses associated with susceptibility to severe COVID-19.

To explore potential intercellular communication mechanisms within this shared signature, we constructed a ligand–receptor interactome by filtering differentially expressed genes using Human Protein Atlas secretome annotation [22] and mapped the resulting secreted ligands to their cognate receptors using the ligand–receptor interaction resource described by Armingol et al. [26]. Communication scores were then calculated according to the expression-based framework proposed by Armingol et al. [26]. Hierarchical clustering of ligand–receptor communication scores identified an interaction module (designated as cluster A in Figure 2D), comprising the secreted ligands lactoferrin (*LTF*), macrophage-stimulating protein 1 (*MST1*), and slit guidance ligand 1 (*SLIT1*), together with their cognate receptors, which consistently exhibited reduced communication scores in individuals with lower susceptibility to severe COVID-19. The coordinated behavior of this module across all three susceptibility-associated comparisons suggested that it represented a conserved intercellular communication program and therefore warranted further investigation. Within cluster A, *LTF* was selected for detailed analysis. Although *LTF* was not the ligand with the highest degree of connectivity in the global interaction network (Figure 2B), it was prioritized because of its well-established role as a mucosal glycoprotein secreted by glandular epithelial cells [40] and its documented antiviral, anti-inflammatory, and immunomodulatory activities against SARS-CoV-2 [39,40,41,42]. Among its predicted candidate receptors (*GPR162*, *LRP1*, and *LRP11*), *LRP1* displayed broad expression across macrophages, consistent with its known role as a low-density lipoprotein receptor (LDLR) family member involved in broader physiological functions and viral host responses [43]. In contrast, *LRP11* stood out among LDLR family members in BALF single-cell analyses due to its marked cell-type-specific enrichment: *LRP11* was expressed in 70% of airway epithelial cells—the primary targets of SARS-CoV-2 infection [18,19]—and 22% of macrophages, while showing minimal expression in other immune populations. This prominent epithelial localization, combined with its viral load-dependent upregulation and reduced expression in patients with low viral load, identified the *LTF*–*LRP11* axis as the most compelling candidate for subsequent analyses.

Although LTF has been widely recognized as a host defense molecule with antiviral and immunomodulatory functions [39,40,41,42], its expression pattern in the present study warrants careful interpretation. Rather than being reduced in groups associated with greater susceptibility to severe COVID-19, *LTF*-associated communication scores were comparatively higher in males, older adults, and patients with high viral load, whereas lower communication scores were consistently observed in individuals with lower susceptibility. This apparent paradox likely reflects the dynamic nature of the host response to SARS-CoV-2 infection. Increased *LTF* expression and signaling may represent a compensatory response to enhanced viral replication, epithelial injury, or inflammation rather than a mechanism directly promoting disease severity. Consequently, our findings suggest that enhanced *LTF*-related communication may represent a transcriptomic marker of an activated mucosal defense response associated with severe disease, rather than evidence of a pathogenic role for LTF itself.

In contrast to the well-characterized biological functions of LTF, comparatively little is known about the role of *LRP11* in respiratory infections. In our analyses, *LRP11* emerged as the most distinctive receptor associated with the LTF signaling axis owing to its pronounced enrichment in airway epithelial cells and its consistent association with viral load. Unlike *LRP1*, which was broadly expressed across macrophages, *LRP11* exhibited a highly restricted cellular distribution, being detected predominantly in epithelial cells, the principal targets of SARS-CoV-2 infection [18,19]. This cell-type-specific localization suggests that epithelial *LRP11* expression may reflect local tissue responses to viral infection rather than generalized immune activation. Although the biological functions of LRP11 remain largely unexplored, its reproducible association with viral burden across independent datasets identifies it as a candidate receptor warranting further functional investigation.

To determine whether these cross-sectional observations reflected dynamic changes during disease progression, we reanalyzed longitudinal BALF transcriptomic data from SARS-CoV-2-infected rhesus macaques [32]. Among the candidate receptors evaluated, *LRP11* exhibited sustained upregulation from day 1 through day 14 post-infection, remaining elevated throughout the course of infection. This temporal pattern is consistent with previous observations that members of the LDLR family participate in host responses to viral infection [44,45]. Importantly, the non-human primate cohort established by Aid et al. was specifically designed to investigate pulmonary vascular disruption, endothelial injury, and microvascular thrombosis following SARS-CoV-2 infection [32]. The progressive increase in *LRP11* expression, together with the enrichment of complement and coagulation pathways identified in our secretome analysis, suggests that epithelial *LRP11* induction occurs in parallel with transcriptomic programs associated with severe COVID-19 progression. Collectively, these findings reinforce the *LTF*–*LRP11* signaling axis as a biologically plausible epithelial communication pathway associated with severe COVID-19 and provide a strong rationale for future mechanistic studies to determine its functional role in disease pathogenesis.

Several limitations of the present study should be considered when interpreting these findings. First, our results are derived from computational analyses of transcriptomic datasets and in silico predictions of ligand–receptor interactions, which infer potential cell–cell communication based on gene expression rather than direct measurements of protein abundance, ligand–receptor binding, or downstream signaling activity. Second, although integrating multiple independent bulk, single-cell, and longitudinal datasets strengthens the robustness and reproducibility of our findings, these publicly available datasets exhibit inherent clinical and methodological heterogeneity, including differences in patient characteristics, sampling time points, and experimental protocols. Finally, while the *LTF*–*LRP11* signaling axis was consistently identified across multiple analytical levels, experimental validation—including protein-level quantification, ligand–receptor binding assays, and functional perturbation studies in airway epithelial models—will be required to establish its biological relevance and determine its mechanistic contribution to severe SARS-CoV-2 infection.

## 5. Conclusions

In conclusion, by integrating transcriptomic data from nasopharyngeal swabs, single-cell BALF profiles, and longitudinal non-human primate infection models, this study identified a convergent secretome signature and highlighted the *LTF*–*LRP11* signaling axis as a candidate epithelial intercellular communication pathway associated with severe COVID-19 susceptibility. The prominent epithelial localization of *LRP11*, together with its association with viral load and sustained expression throughout infection, supports its potential involvement in epithelial responses during disease progression. Beyond prioritizing the *LTF*–*LRP11* axis for future functional investigation, these findings demonstrate the utility of secretome-centric interactome profiling for identifying candidate intercellular signaling pathways underlying respiratory viral diseases.

## Figures and Tables

**Table 1:** Comparisons for each differential expression analysis and between COVID-19 patients and SARS-CoV-2-negative individuals, with their respective numbers of individuals (*n*).

Comparisons	Name
COVID-19 (*n* = 430) × Negative individuals (*n* = 54)	COVID-19
Positive female (*n* = 201) × Negative female (*n*= 30)	Female
Positive male (*n* = 176) × Negative male (*n* = 24)	Male
Positive young (*n* = 241) × Negative young (*n* = 39)	Young
Positive old (*n* = 172) × Negative old (*n* = 15)	Old
Low viral load (*n* = 99) × Negative individuals (*n* = 54)	Low viral load
High viral load (*n* = 108) × Negative individuals (*n* = 54)	High viral load

## Data Availability

The data presented in this study are available in the Gene Expression Omnibus (GEO) repository at https://www.ncbi.nlm.nih.gov/geo/, reference numbers GSE152075, GSE145926, GSE128033 (sample GSM3660650), and GSE156701.

## Supplementary Materials

The following supporting information can be downloaded at https://www.mdpi.com/article/10.3390/genes17080982/s1, Figure S1. Transcriptomic analyses of nasopharyngeal swab samples from COVID-19-positive patients in the GSE152075 dataset. (A) Total number of COVID-19-positive and -negative samples in their respective groups: COVID-19, gender (women and men), age (young, <60 years; elderly, ≥60 years), and viral load (low and high). (B) Heatmap of logFC expression values for each analyzed comparison. Rows (secretome genes) and columns (comparisons) were clustered using k-means. (C) UpSet plot of NP swab sample secretome DEGs shared between comparisons. Figure S2. Volcano plots of DEGs from the secretome of NP swab samples from COVID-19 patients, showing altered expression across comparisons (COVID-19, gender, age, and viral load). Each point represents a single gene. Red points represent genes with increased expression, whereas blue points represent genes with decreased expression. (A) COVID-19. (B) Women. (C) Men. (D) Young individuals. (E) Elderly individuals. (F) Low viral load. (G) High viral load. Figure S3. Bar plot illustrating the percentage expression of each ligand and receptor within cluster A across various cell types in BALF samples. Data were retrieved from the GSE145926 dataset, along with the healthy BALF sample (GSM3660650) from the GSE128033 dataset, and analyzed using the Seurat package (v. 3) in R. Figure S4. Dot plot depicting the expression levels of genes belonging to the LDL receptor family in each cell type identified in BALF samples. Data were retrieved from the GSE145926 dataset and the healthy BALF sample (GSM3660650) from the GSE128033 dataset and analyzed using the Seurat package (v. 3) in R. Table S1. Raw data used for analysis (GSE152075—https://www.ncbi.nlm.nih.gov/geo/query/acc.cgi?acc=GSE152075, accessed on 1 March 2021). Table S2. List of 2641 secretome genes, available from The Human Protein Atlas (https://www.proteinatlas.org/humanproteome/secretome, accessed on 1 March 2021). Table S3. Transcriptional profile of post-secretome filtering COVID-19 comparison. Table S4. Transcriptional profile of post-secretome filtering female comparison. Table S5. Transcriptional profile of post-secretome filtering male comparison. Table S6. Transcriptional profile of post-secretome filtering comparison of young patients. Table S7. Transcriptional profile of post-secretome filtering comparison of old patients. Table S8. Transcriptional profile of post-secretome filtering low-viral-load comparison. Table S9. Transcriptional profile of post-secretome filtering high-viral-load comparison. Table S10. Characterization of the 43 unique genes of the less-severe-COVID-19 group. Table S11. Raw data used to assemble the time course (GSE156701—https://www.ncbi.nlm.nih.gov/geo/query/acc.cgi?acc=GSE156701, accessed on 30 June 2023).
